# Comparison of radiological interpretation made by veterinary radiologists and state-of-the-art commercial AI software for canine and feline radiographic studies

**DOI:** 10.3389/fvets.2025.1502790

**Published:** 2025-02-21

**Authors:** Yero S. Ndiaye, Peter Cramton, Chavdar Chernev, Axel Ockenfels, Tobias Schwarz

**Affiliations:** ^1^Department of Economics, University of Cologne, Cologne, Germany; ^2^Max Planck Institute for Research on Collective Goods, Bonn, Germany; ^3^Department of Economics, University of Maryland, Baltimore, MD, United States; ^4^Royal (Dick) School of Veterinary Studies and Roslin Institute, The University of Edinburgh, Edinburgh, United Kingdom; ^5^Department for Small Animal Medicine & Surgery, University of Veterinary Medicine Hannover, Hannover, Germany

**Keywords:** veterinary diagnostic imaging, artificial intelligence, machine learning, dog, cat

## Abstract

**Introduction:**

As human medical diagnostic expertise is scarcely available, especially in veterinary care, artificial intelligence (AI) has been increasingly used as a remedy. AI's promise comes from improving human diagnostics or providing good diagnostics at lower cost, increasing access. This study analyzed the diagnostic performance of a widely used AI radiology software vs. veterinary radiologists in interpreting canine and feline radiographs. We aimed to establish whether the performance of commonly used AI matches the performance of a typical radiologist and thus can be reliably used. Secondly, we try to identify in which cases AI is effective.

**Methods:**

Fifty canine and feline radiographic studies in DICOM format were anonymized and reported by 11 board-certified veterinary radiologists (ECVDI or ACVR) and processed with commercial and widely used AI software dedicated to small animal radiography (SignalRAY^®^, SignalPET^®^ Dallas, TX, USA). The AI software used a deep-learning algorithm and returned a coded *abnormal* or *normal* diagnosis for each finding in the study. The radiologists provided a written report in English. All reports' findings were coded into categories matching the codes from the AI software and classified as *normal* or *abnormal*. The sensitivity, specificity, and accuracy of each radiologist and the AI software were calculated. The variance in agreement between each radiologist and the AI software was measured to calculate the ambiguity of each radiological finding.

**Results:**

AI matched the best radiologist in accuracy and was more specific but less sensitive than human radiologists. AI did better than the median radiologist overall in low- and high-ambiguity cases. In high-ambiguity cases, AI's accuracy remained high, though it was less effective at detecting abnormalities but better at identifying normal findings. The study confirmed AI's reliability, especially in low-ambiguity scenarios.

**Conclusion:**

Our findings suggest that AI performs almost as well as the best veterinary radiologist in all settings of descriptive radiographic findings. However, its strengths lie more in confirming normality than detecting abnormalities, and it does not provide differential diagnoses. Therefore, the broader use of AI could reliably increase diagnostic availability but requires further human input. Given the unique strengths of human experts and AI and the differences in sensitivity vs. specificity and low-ambiguity vs. high-ambiguity settings, AI will likely complement rather than replace human experts.

## 1 Introduction

Human medical diagnostics can be prone to error, with bias leading to systematically skewed judgments and noise, causing errors due to random variations in judgment. Numerous strategies and protocols have been adopted in medical services to mitigate these issues (1). A rapidly growing and promising remedy is Artificial Intelligence (AI). Methods such as neural networks and autoencoders, which excel in medical image recognition, show significant promise for radiology (2–5). Studies have demonstrated that AI can effectively recognize abnormalities in various radiological image-recognition tasks (6) and often performs at least as well as well-trained and experienced radiologists (7).

To date, these studies have primarily focused on human radiology (8). AI has been successfully implemented in human medicine and is now gaining traction in veterinary medicine (9, 10). This development is particularly relevant given the scarcity of experts and their limited availability, which restricts access to medical diagnostics in veterinary medicine, especially diagnostic imaging. The American and European radiology board specialist organizations have <2,000 members to serve the entire European and North American continents. Therefore, it is reasonable to question whether the proliferation of radiological AI could improve outcomes in veterinary healthcare. Firstly, if AI performs no worse than human radiologists, it can help increase the availability of diagnostics. Secondly, if AI performs as well as humans but errs differently from humans, effective protocols may leverage the strengths of both. Thirdly, AI could lead to better diagnostics and, thus, better overall health outcomes. If AI is more accurate, it could reduce bias. Additionally, AI is likely to exhibit less random variation than an equally accurate human expert, improving outcomes by reducing or eliminating noise.

The purpose of this study was to compare the diagnostic performance for the interpretation of canine and feline radiographs of a commonly used commercial and proprietary AI radiology software to the performance of veterinary radiologists. We hypothesized that (1) the diagnostic performance of AI radiology software would be at least as good as that of the median human radiologist, and (2) the diagnostic performance of AI radiology software would be worse relative to the median human radiologists in high-ambiguity settings.

## 2 Material and methods

### 2.1 Study and dataset selection and observers

A retrospective diagnostic performance study was undertaken, and the CLAIM guidelines were followed (11). Each radiograph was evaluated by veterinary radiologists and the AI software, which we call *observers*. Fifty canine and feline radiographic studies in DICOM format were randomly retrieved and anonymized from the institutional PACS. There were forty canine and ten feline radiographic studies. The studies included the complete thoracic cavity in 23 cases and the entire abdominal cavity in 11 cases, and they focused on the musculoskeletal system in 26 cases. Radiographic studies with non-diagnostic quality or those taken for implant and stent checks were excluded. One co-author (CC) reviewed the retrieved cases for compliance with the inclusion criteria and supplemented the imaging data with signalment data (age, species, breed, sex, and neutered status) and a short clinical history and purpose of the study. These data were retrieved from each study's diagnostic imaging request form stored in the patient management system. The hospital's caseload consisted of primary and referred patient veterinary care.

### 2.2 AI software

All 50 anonymized DICOM studies were processed with commercial AI software dedicated to small animal radiography (SignalRAY^®^, SignalPET^®^ Dallas, TX, USA). The software is continuously updated and does not have version numbers; it was accessed in July 2022. SignalRAY is a useful benchmark for AI software available in practice, as it is used by over 2,300 clinics worldwide and interprets around 50,000 radiographs per week. It is used in roughly 5% of US practices and almost 2% worldwide.

#### 2.2.1 Deep-learning architecture

Since SignalRAY^®^ employs a proprietary algorithm, full architectural details are unavailable. However, key features relevant to understanding the algorithm's functionality can be presented. SignalRAY is applied to various binary and multiclass computer vision tasks, including image-level classification, segmentation, localization, and study-level classification. It utilizes 38 different deep-learning architectures. Most computer vision models consist of convolutional neural networks (CNNs) with multiple intermediate layers for hierarchical feature extraction and classification (12). Head networks and skip connections are incorporated to preserve fine-grained spatial information. The CNNs include ConvNeXt variants (13), which enhance feature extraction through their convolutional designs; the EfficientNetfamily (14), selected for their balance of accuracy and computational efficiency; ResNet architectures (15), employed for their ability to train very deep networks effectively; and U-Net and its derivatives (16), primarily used for segmentation tasks.

#### 2.2.2 Model training and evaluation

SignalRAY^®^ was trained on a large, multicentric dataset comprising radiographic images and clinical data from over 3,000 veterinary practices across more than ten countries. The dataset was designed to broadly represent varied clinical settings, patient populations, and imaging equipment. The training set size for each model typically ranged from 10,000 to 60,000 images.

A stratified train-test-validation split method was implemented to maintain independence between the datasets and to ensure robustness in evaluation across different conditions, animal species, and image quality. Data partitioning typically allocated 70% for training, 15% for validation, and 15% for testing.

During training, several models were optimized for each task. Optimization included data augmentation techniques such as geometric transformations (rotations, flips, scaling), intensity adjustments (brightness, contrast), noise injection (Gaussian, speckle), and domain-specific augmentations (e.g., artifact introduction). Additionally, the high-dimensional hyperparameter space, including learning rates, batch sizes, regularization strengths, and architectural choices (e.g., depth and width of networks), was optimized. The models were further refined, and the final model for each task was selected based on standard performance metrics such as accuracy, area under the receiver operating characteristic, and precision-recall curves. Further criteria were error analysis based on the expected clinical impact according to veterinary experts, interpretability of results, and time stability (17).

#### 2.2.3 Model output

The software processed only the images provided without considering additional clinical information. Based on the information from all radiographs in the study, it provided a coded diagnosis, normal or abnormal, for each case. The outcome data from the processed studies were formatted into codes corresponding to specific radiographic findings, and these coded diagnostic findings and diagnoses were recorded.

### 2.3 Human veterinary radiologists

**Figure 1:**
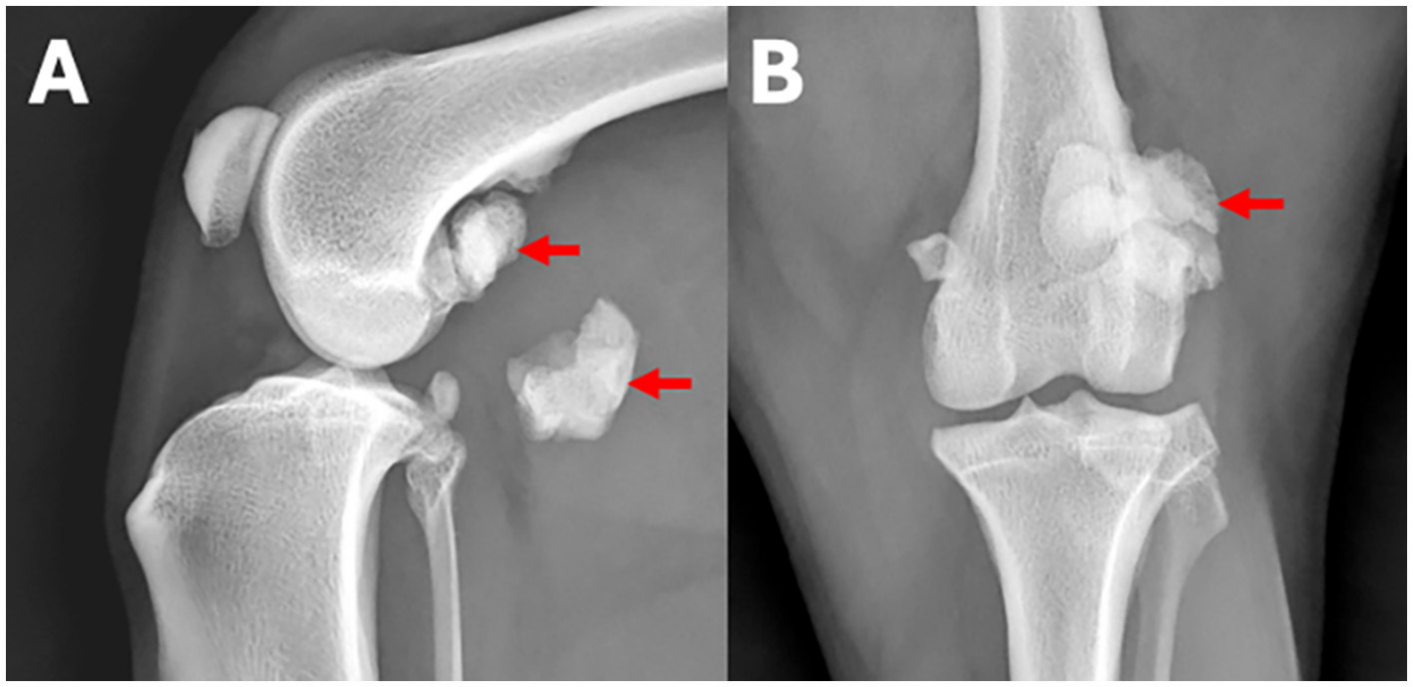
**(A)** Lateral and **(B)** craniocaudal radiographs of the left stifle region of a 7-year-old Border collie with left hind lameness. An example for a human observer statement classified as a normal finding is: *Both stifle joints are congruent*. An example for an observer statement classified as abnormal is: *The lateral fabella is enlarged with regular margins and fragmented* (arrows).

Board-certified veterinary radiologists (ECVDI or ACVR) were recruited via advertisements on the ACVR and ECVDI websites and mailing lists. Participating radiologists were given PACS access to the anonymized cases, patient signalment, short history, and clinical signs. They were asked to provide a written report in English in digital format. The report should contain a descriptive and a diagnostic part and be written in the style the radiologist was used to reporting. The complete reports from veterinary radiologists were recorded and manually coded into categories that matched the codes from the AI software. All findings were also classified as *normal* or *abnormal* (Figure 1). Findings where radiologists implied a normal variation were coded as normal. On the other hand, findings where radiologist implied insignificant abnormality were considered abnormal. If a radiologist or the AI did not mention an observation made by others, it was assumed that this radiologist or AI considered the observation normal. The differential diagnoses veterinary radiologists gave were not investigated as the AI software did not provide any comparable outcome.

### 2.4 Ground truth and performance metrics

To assess the diagnostic performance of each observer *i*, the true state of each finding must first be established. Consider finding *f*_*j,k*_ with *j* being the radiograph and *k* being the finding in the radiograph. Each finding is either abnormal (*f*_*j,k*_ = 1) or normal (*f*_*j,k*_ = 0). To proxy the true state of each finding the *consensus* of all observers ${\hat{f}}_{j,k}$was taken. Considering the assessment of each observer ${\hat{f}}_{j,k,i}$, the consensus is:


$$\begin{array}{l} {\hat{f}}_{j,k}=𝕀\left(\frac{1}{n}\sum_{i=1}^{n}{\hat{f}}_{j,k,i}\geq 0.5\right),\;n\geq 5. \end{array}$$

If the median observer regarded the finding as normal, it was considered normal and abnormal otherwise. Therefore, for each finding *f*_*j,k*_ evaluated by observer *i*, there were four potential outcomes: true positive (*TP*_*j,k,i*_), true negative (*TN*_*j,k,i*_), false positive (*FP*_*j,k,i*_), and false negative (*FN*_*j,k,i*_).

Three commonly used metrics—accuracy, sensitivity, and specificity—were used to evaluate each observer's diagnostic quality.


$$\begin{array}{l} \;Accuracy_{i}=\frac{\sum_{j}\sum_{k}TN_{j,k,i}+TP_{j,k,i}}{\sum_{j}\sum_{k}TN_{j,k,i}+TP_{j,k,i}+FN_{j,k,i}+FP_{j,k,i}},\\ Sensitivity_{i}=\frac{\sum_{j}\sum_{k}TP_{j,k,i}}{\sum_{j}\sum_{k}TP_{j,k,i}+FN_{j,k,i}},\\ Specificity_{i}=\frac{\sum_{j}\sum_{k}TN_{j,k,i}}{\sum_{j}\sum_{k}TN_{j,k,i}+FP_{j,k,i}}. \end{array}$$

The observers' metrics were compared using a *z*-test for proportions. As all radiologists were compared to the AI for each metric, a Bonferroni correction for multiple hypotheses testing was applied. Adjusted *p*-values smaller than 0.05 were considered as significant.

To further allow an investigation into whether certain situations favored radiologists vs. the AI, we calculated the inter-observer agreement level of each radiological finding by establishing the variance of assessment (*V*) for each finding:


$$\begin{array}{l} V_{j,k}=\frac{1}{n}\sum_{i=1}^{n}{\left({\hat{f}}_{j,k,i}-f_{j,k}\right)}^{2}. \end{array}$$

Findings in which all observers agreed had a variance of zero while findings where half the observers assessed abnormal (*f*_*j,k*_ = 1) and half assessed normal (*f*_*j,k*_ = 0) had a variance of 0.3. Observations without unanimity in agreement and below the median variance were considered as *low ambiguity*, as it implies an easier to detect true state. Observations without unanimity in agreement and above the median variance were considered as *high ambiguity*. Then the same metrics for each situation were computed, and the z-test was used for proportions to assess whether there were differences in performance between the AI and radiologists in each situation.

## 3 Results

The 50 radiographic studies had a mean of 5.64 radiographs, with a range of 1 to 16 radiographs per study. Eleven veterinary radiologists participated in the study, each providing a mean of 24.8 radiologic reports (range 24–25), resulting in a mean of 5.46 reports per study and a total of 16,434 radiological findings. Consensus established an imbalanced sample, with 84% of findings being normal and 16% being abnormal.

For 47% of these findings, there was unanimous agreement among all radiologists and the AI software. For findings without unanimity, there were 2,546 observations with low ambiguity and 3,370 observations with high ambiguity. The rate of normal findings decreased in the absence of unanimity and the presence of high ambiguity. The distribution of the sample is summarized in Table 1.

**Table 1:** Distribution of normal and abnormal findings for different subsamples.

**Sample**	**All**	**No unanimity**	**Low ambiguity**	**High ambiguity**
Normal (%)	83.89	79.53	90.97	75.40
Abnormal (%)	16.11	20.47	9.03	24.60
Total number of findings	16,434	8,646	2,546	3,370

### 3.1 Aggregated results

**Figure 2:**
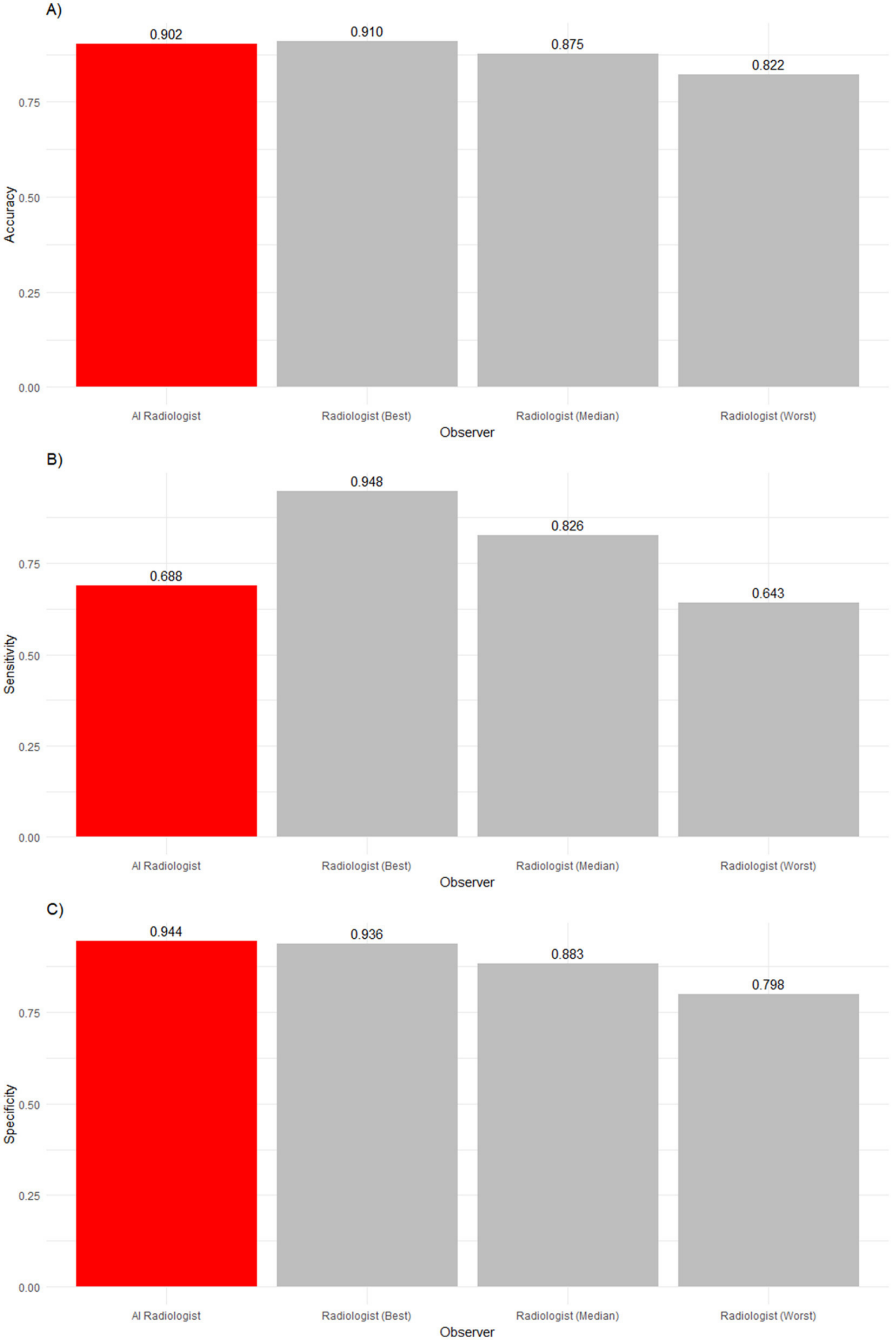
**(A)** Accuracy, **(B)** sensitivity, and **(C)** specificity of an AI radiologist (red bar), best, median, and worst performing human radiologists (gray bars) for all findings. The AI accuracy and specificity were higher than those of the median radiologist, but the sensitivity was lower.

The diagnostic results are displayed in Table 2. Further, the worst-performing, median-performing, and best-performing radiologists, as well as the AI software for all studies with all findings, are displayed in Figure 2. The worst-performing, median-performing, and best-performing radiologists (henceforth worst, median, and best radiologists) were re-determined for each metric, meaning the best and worst radiologists may vary across different metrics. Thus, they represented an upper and lower benchmark in all cases. To benchmark the performance, recall that the prevalence of normal findings in our data was 84%. Thus, a radiologist probabilistically guessing “normal” for every case would achieve an accuracy of 84%. The accuracy of the median and best radiologist was significantly higher than probabilistic guessing while the performance of the worst radiologist was not significantly different from probabilistic guessing. The AI software was significantly more accurate than any median radiologist (*p* = 0.0022). There was no significant difference in performance between the best radiologist and the AI software (*p* = 0.0561). Thus, the AI software was no less accurate than any radiologist, confirming our first hypothesis.

**Table 2:** Accuracy, sensitivity, and specificity by observer for all findings.

**Observer**	**Accuracy**	**Sensitivity**	**Specificity**
R1	0.910	0.779^***^	0.936^***^
*AI Software*	*0.902*	*0.688*	*0.944*
R2	0.898	0.784^***^	0.921^***^
R3	0.889	0.643	0.926
R4	0.886	0.824^***^	0.899^***^
R5	0.882	0.824^***^	0.899^***^
R6	0.875^***^	0.860^***^	0.878^***^
R7	0.867^***^	0.784	0.883^***^
R8	0.862^***^	0.865^***^	0.861^***^
R9	0.860^***^	0.898^***^	0.852^***^
R10	0.856^***^	0.826^***^	0.861^***^
R11	0.822^***^	0.948^***^	0.798^***^

* p < 0.1,

** p < 0.05,

*** p < 0.01 (based on Bonferroni-corrected Proportion tests for difference between Human observer and AI software).

The frequency of abnormalities in our sample was 16%. Thus, the sample was imbalanced. The performance in detecting abnormalities varied widely. While the worst radiologist detected only slightly more than six in 10 abnormalities, the median radiologist detected more than eight in 10 abnormalities, and the best radiologist detected 92% of abnormalities. Human radiologists tended to be much better than the AI software (68.8%) in detecting abnormalities, with nine radiologists being significantly more sensitive (*p* < 0.0001). Two radiologists' sensitivity did not significantly differ from the AI software (*p* = 0.497 and *p* > 0.9999).

In the analysis of observers' abilities to detect normality, a reverse picture compared to sensitivity was found. The AI software detected 94.4% of all normal findings, whereas the best human radiologist detected only 93.6% of normal findings, the median human radiologist detected 88.3% of normal findings, and the worst human radiologist detected 79.8% of normal findings. The AI software significantly outperformed ten radiologists (*p* < 0.0001). However, one veterinary radiologist was as good as the AI software in identifying normality (*p*> 0.9999).

Table 3 displays the results for findings with no unanimity among observers. There, accuracy, sensitivity, and specificity declined for all observers. While the differences in sensitivity and specificity between AI and veterinary radiologists remained unchanged, the best veterinary radiologist significantly outperformed the AI in accuracy (*p* = 0.0352). However, the AI still significantly outperformed all below-median radiologists (*p* ≤ 0.0011).

**Table 3:** Accuracy, sensitivity, and specificity by observer for non-unanimous findings.

**Observer**	**Accuracy**	**Sensitivity**	**Specificity**
R1	0.826^**^	0.677^***^	0.868^***^
*AI Software*	*0.814*	*0.533*	*0.887*
R2	0.804	0.684^***^	0.837^***^
R4	0.781	0.743^***^	0.792^***^
R5	0.773	0.789^***^	0.769^***^
R6	0.759^**^	0.796^***^	0.749^***^
R3	0.750	0.500	0.816
R7	0.750^***^	0.672	0.769^***^
R8	0.740^***^	0.795^***^	0.726^***^
R9	0.729^***^	0.851^***^	0.696^***^
R10	0.729^***^	0.735^***^	0.727^***^
R11	0.665^***^	0.922^***^	0.601^***^

* p < 0.1,

** p < 0.05,

*** *p* < 0.01 (based on Bonferroni-corrected Proportion tests for the difference between Human observer and AI software).

### 3.2 Results by level of ambiguity

**Figure 3:**
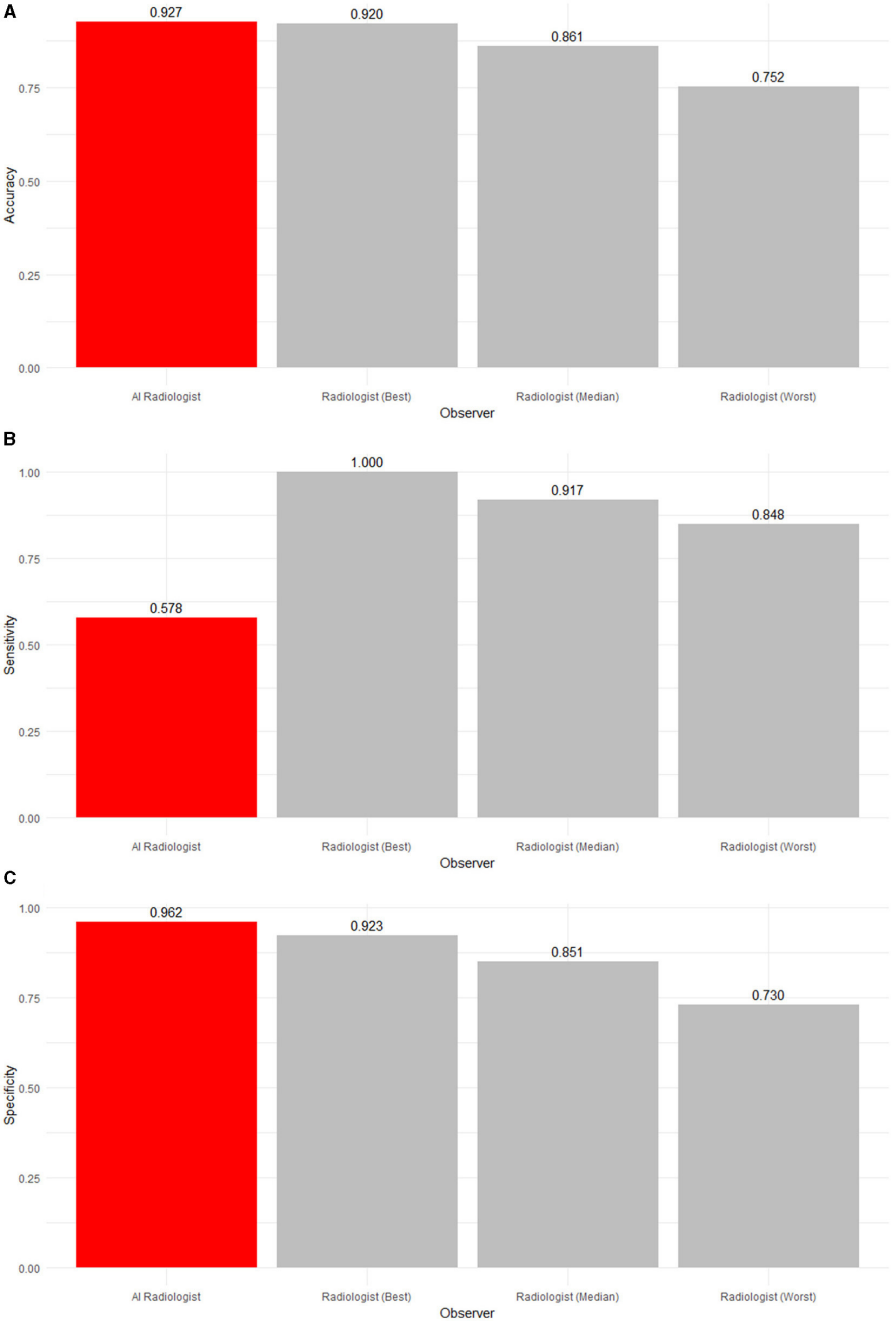
**(A)** Accuracy, **(B)** sensitivity, and **(C)** specificity of an AI radiologist (red bar), best, median, and worst performing human radiologists (gray bars) for low-ambiguity findings. The AI software had the highest accuracy and specificity but the lowest sensitivity.

**Figure 4:**
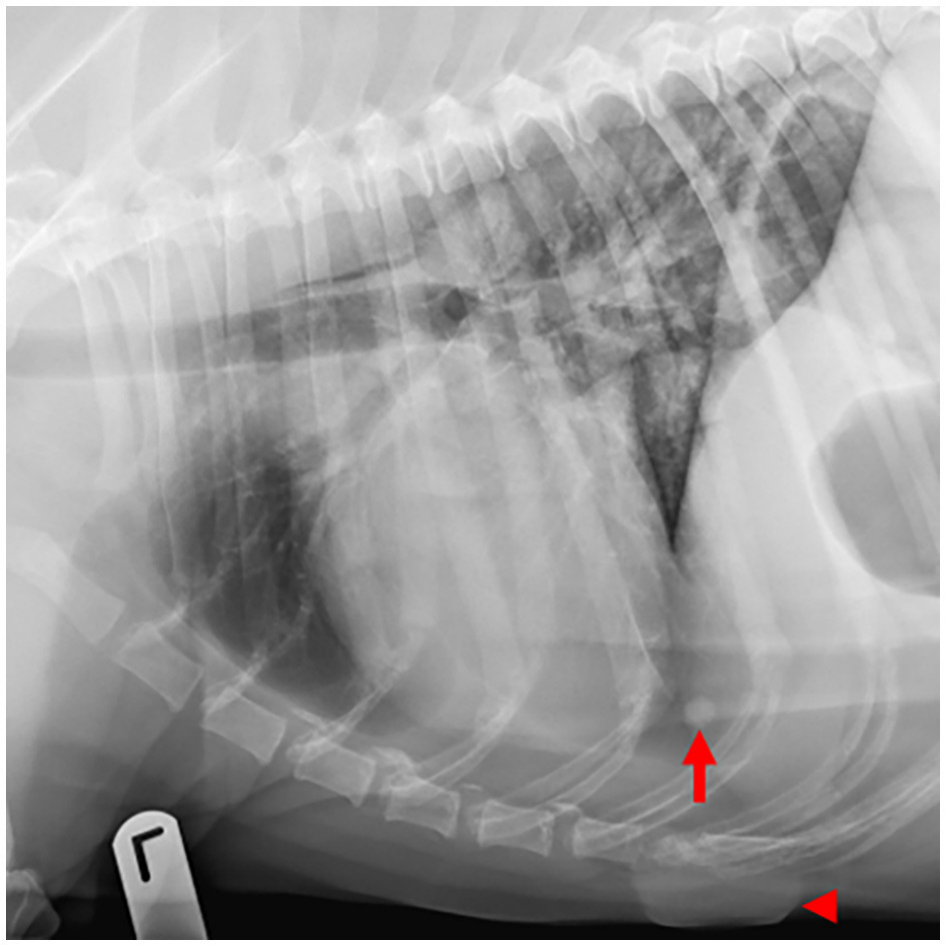
Example of a case with low-ambiguity radiographic findings. On this thoracic radiographic study of a 12-year-old Labrador retriever presenting for routine staging of a digital melanoma, all 8 human observers and the AI software identified pulmonary nodules (arrow) and a skin mass (arrowhead).

Table 4 and Figures 3, 4 summarize and illustrate the diagnostic performance in the low ambiguity situation. The worst radiologist diagnosed worse than probabilistic guessing (accuracy of 0.752), while the median (accuracy of 0.861) and best radiologist (accuracy of 0.920) as well as the AI software (accuracy of 0.927) were significantly better. The AI software had the highest accuracy and was significantly more accurate than eight radiologists (*p* ≤ 0.0231) and as accurate as three radiologists (*p* ≥ 0.294). However, the AI software was the least sensitive (0.578) in detecting abnormalities (*p* < 0.0001). However, four out of 11 radiologists did not have low-ambiguity abnormality in the list of studies they interpreted. Therefore, it could not be evaluated how the AI software comparatively performs. In low ambiguity situations, the AI software was again the most specific (0.962) in detecting normality. Indeed, it was significantly more specific than any radiologist (*p* ≤ 0.0231).

**Table 4:** Accuracy, sensitivity, and specificity by observer for low-ambiguity findings.

**Observer**	**Accuracy**	**Sensitivity**	**Specificity**
*AI-Software*	*0.927*	*0.578*	*0.962*
R1	0.920	0.900^***^	0.923^***^
R2	0.902	0.848^***^	0.910^***^
R10	0.889^**^	-	0.889^**^
R8	0.889^**^	-	0.889^**^
R7	0.889^**^	-	0.889^**^
R5	0.864^***^	0.917^***^	0.857^***^
R6	0.861^***^	0.939^***^	0.851^***^
R4	0.837^***^	0.891^***^	0.831^***^
R3	0.762	1.000^***^	0.737^***^
R9	0.752^***^	0.926^***^	0.730^***^
R11	0.611^***^	-	0.611^***^

* p < 0.1,

** *p* < 0.05,

*** *p* < 0.01 (based on Bonferroni-corrected Proportion tests for the difference between Human observer and AI software). The sensitivity for R7, R8 and R10 could not be computed as there was no low-ambiguity abnormality for any study they rated.

**Figure 5:**
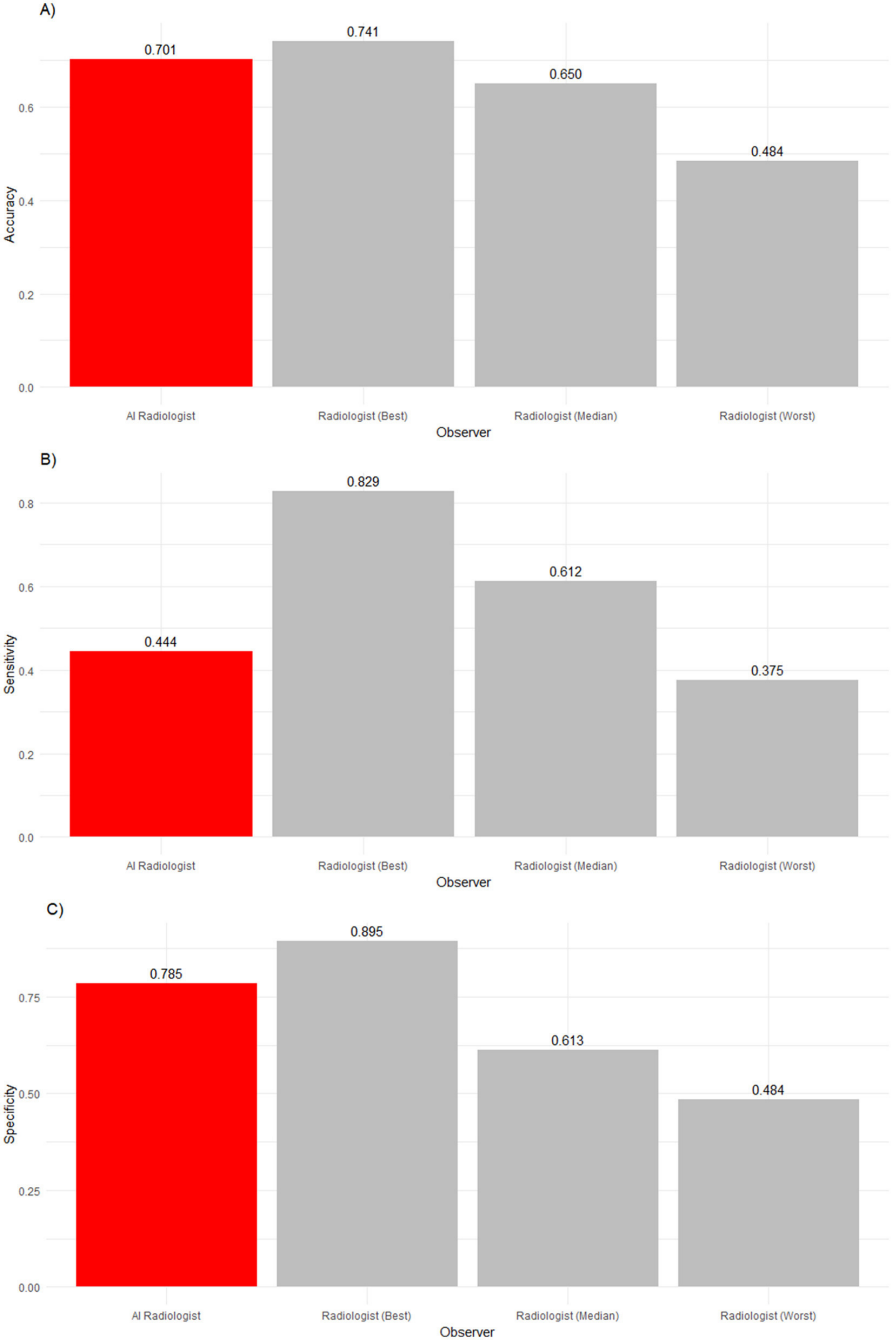
**(A)** Accuracy, **(B)** sensitivity and **(C)** specificity of an AI radiologist (red bar), best, median and worst performing human radiologists (gray bars) for high-ambiguity findings. Compared to the median radiologist, the AI accuracy and specificity was higher, but the sensitivity was lower.

**Figure 6:**
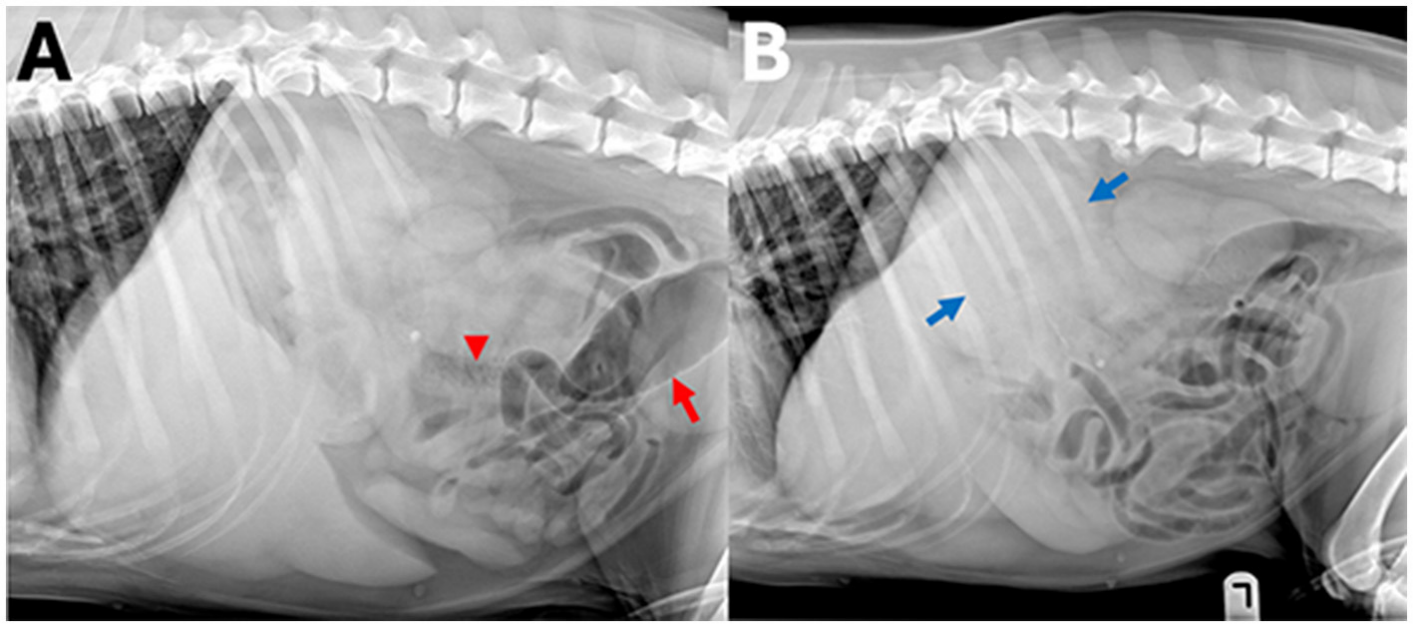
Example of a case with high-ambiguity radiographic findings. In this abdominal radiographic study [**(A)** right lateral, **(B)** left lateral radiograph] of a 7-year-old Golden retriever with stage 5 lymphoma presenting for vomiting. A wide range of radiographic findings were identified by human observers, including a perisplenic soft tissue opacity or mass (observer 1, 2, and 5, blue arrows), small intestinal granular material (observer 1, 3, and 5, arrowhead), and colonic gas distension (observers 1, 2, and 5, red arrow), whereas observer 4 and the AI observer did not report any of these findings.

Table 5 and Figures 5, 6 summarize and illustrate the diagnostic performance in the high ambiguity situation. Accuracy decreased for the worst (0.484), median (0.650), and best radiologists (0.741), as well as the AI software (0.701). The AI software was significantly more accurate than seven radiologists (*p* ≤ 0.0011) but insignificantly less accurate than the best radiologist (*p* = 0.807) refuting our second hypothesis. The AI did not detect abnormalities as well as seven radiologists (*p* ≤ 0.0022). In terms of specificity, the AI performed significantly better than all but two radiologists (*p* ≤ 0.0198) but significantly worse than the best radiologist (*p* = 0.0011).

**Table 5:** Accuracy, sensitivity, and specificity by observer for high-ambiguity findings.

**Observer**	**Accuracy**	**Sensitivity**	**Specificity**
R3	0.741	0.375	0.895^***^
*AI Software*	*0.701*	*0.444*	*0.785*
R4	0.700^***^	0.677^***^	0.713^**^
R9	0.695^***^	0.817^***^	0.623^***^
R1	0.687^***^	0.579^***^	0.75
R2	0.657	0.612^***^	0.683^***^
R5	0.650	0.733^***^	0.601^***^
R6	0.610	0.733^***^	0.537^***^
R7	0.605^***^	0.563	0.613^***^
R10	0.556^***^	0.602^***^	0.547^***^
R11	0.541^***^	0.828^***^	0.487^***^
R8	0.484^***^	0.482^***^	0.484^***^

* *p* < 0.1,

** *p* < 0.05,

*** *p* < 0.01 (based on Bonferroni-corrected Proportion tests for the difference between Human observer and AI software).

## 4 Discussion

In this study, the AI software performed in parts on par with the best radiologist in reporting radiographic findings and was at least better than the median radiologist, confirming our first hypothesis. The broader use of AI could reliably increase the availability of diagnostic expertise, particularly in settings where human radiologists are scarce. The AI strengths were more pronounced in confirming normality than detecting abnormal findings, and it performed better in a low-ambiguity situation. Veterinarians dealing with AI-generated radiographic interpretation results should consider this. The relevance of false positive or false negative findings depends on the clinical circumstances of the case, so diagnostic results should always be interpreted in that context. The high-ambiguity settings captured cases where radiographic findings were most nuanced and required the most clinical context. Our hypothesis that human radiologists would outperform AI in accuracy in a high ambiguity situation assumed that the plasticity of human reasoning would be better suited for this than a mechanistic AI pattern recognition based on a finite training set, but this was rejected in this study. We suspect that the increased variance reflects the increased inter-observer noise of human radiologists, where different observers vary in their judgment of the same problem. AI software also showed decreased accuracy in these circumstances. AI software may be most effective as a complementary tool rather than a replacement for human experts. Diagnostic expertise may matter more for difficult findings, while simpler findings could be entirely outsourced. Potentially, AI could be used to pre-screen radiographs expected to be low ambiguity. This could streamline the diagnostic process, allowing radiologists to focus on more complex cases and potentially improving diagnostic accuracy and efficiency. Integrating AI into the clinical workflow could have important practical implications but is likely not straightforward. Few studies have empirically evaluated the nature of collaboration between human healthcare professionals and AI (18). Some found that “good” AI improved the decision-making of physicians. This effect was most pronounced for the least experienced physicians. However, they also found that “faulty” AI can mislead physicians, including experts (18). Moreover, a recent large study found that AI often made radiologists' performance worse and that optimal human-AI collaboration was achieved by delegating cases to humans or AI but not AI-assisted humans (19). Therefore, ensuring good protocols on how AI is used to complement the work of radiologists, potentially reducing their workload and improving overall efficiency in diagnostic practices, is crucial.

There were several limitations of this study. Our study investigated only a relatively small sample of radiographic studies with two species and multiple body parts depicted. This was mainly due to funding and resource restrictions and is similar to other AI studies in the veterinary field (20–22). Our radiographic studies were imbalanced as most findings were normal. Our results regarding accuracy would be biased if this imbalance would not reflect the distribution in practice. However, our findings regarding sensitivity and specificity would not be affected. Our results depended on the selection of observers. As the AI rated more radiographic studies than each of the radiologists individually, the estimates of its performance may be marginally more precise although no bias in the estimates was introduced. Several AI systems are available, and we chose a state-of-the-art, market-relevant 2022 version software. It is reasonable to assume that AI software has improved over time, making our findings a lower benchmark for the comparative performance of AI available today. We recruited radiologists via advertisements which could have introduced a selection bias. Any study of diagnostic performance requires defining the underlying true state. Our analysis opted for a consensus approach, leveraging the wisdom of the crowd rather than relying on any single expert or other means of confirmation. However, this consensus may sometimes be incorrect, especially in high-ambiguity cases, when human inter-observer noise is likely highest. There is no objective way to classify the relevance of descriptive findings. Reporting any irrelevant but obviously incidental finding increases the total number of findings and thus affects our estimates of accuracy and specificity. Nevertheless, since we compared observers' performance relatively, this did not impact our assessment of how well AI performs relative to human radiologists. However, the absolute point estimates should be taken with a grain of salt. We limited our study to descriptive findings and did not consider differential diagnoses. This limitation was necessary because the AI software we evaluated did not provide differential diagnoses. This exclusion of differential diagnoses may have led to an underestimation of the human radiologists' overall performance. Differential diagnoses add important nuances to the report and give the receiving veterinarian a broader picture of the case. Future research should address these limitations by incorporating differential diagnoses, improving observer selection methods, and continuously updating the AI software to ensure its relevance.

## 5 Conclusion

State-of-the-art commercial veterinary radiology AI can be used reliably to determine descriptive findings from canine and feline radiographs, making it suitable for broader use and increasing the availability of expertise in the field. While AI shows promise in augmenting veterinary diagnostic capabilities, its optimal use likely lies in complementing human expertise. By leveraging the strengths of AI and human radiologists, AI can improve diagnostic accuracy, increase the availability of expert opinions, and enhance patient outcomes in veterinary healthcare.

## Data Availability

The original contributions presented in the study are included in the article, further inquiries can be directed to the corresponding author.
